# Prevalence of Trachoma in Car-Nicobar Island, India after Three Annual Rounds of Mass Drug Administration with Azithromycin

**DOI:** 10.1371/journal.pone.0158625

**Published:** 2016-07-08

**Authors:** Sumit Malhotra, Praveen Vashist, Noopur Gupta, Mani Kalaivani, Gita Satpathy, Anita Shah, Sujaya Krishnan, Rajvardhan Azad

**Affiliations:** 1 Department of Community Ophthalmology, Dr. Rajendra Prasad Centre for Ophthalmic Sciences, All India Institute of Medical Sciences, New Delhi, India; 2 Department of Biostatistics, All India Institute of Medical Sciences, New Delhi, India; 3 Department of Ocular Microbiology, Dr. Rajendra Prasad Centre for Ophthalmic Sciences, All India Institute of Medical Sciences, New Delhi, India; 4 Gobind Ballabh Pant Hospital, Port Blair, Andaman and Nicobar Islands, India; 5 Ministry of Health and Family Welfare, Government of India, New Delhi, India; 6 Dr. Rajendra Prasad Centre for Ophthalmic Sciences, All India Institute of Medical Sciences, New Delhi, India; Istituto Nazionale Tumori, ITALY

## Abstract

**Background:**

A high proportion of active trachoma infection in children of Car-Nicobar Island was reported through the Trachoma Rapid Assessment survey conducted in year 2010 by the same researchers. Annual mass drug treatment with azithromycin was administered from years 2010–12 to all individuals residing in this island for reducing the burden of active trachoma infection. A cross-sectional prevalence survey was conducted in the year 2013 to assess the post-treatment burden of trachoma in this population.

**Methods:**

In the 15 randomly selected compact segments from each village of the island, children aged 1–9 years were examined for evidence of active trachoma infection and participants aged ten years and above were examined for trachomatous trichiasis and corneal opacity.

**Results:**

A total of 809 children (1–9 years) and 2735 adults were examined. Coverage with azithromycin for all the three rounds was more than 80%. The prevalence of active trachoma infection in children aged 1–9 years old was 6.8% (95% CI 5.1, 8.5) and Trachomatous Trichiasis (TT) was 3.9% (95% CI 3.2, 4.6). The risk factors associated with active trachoma infection were older age and unclean faces. The risk factors associated with TT were older age and lower literacy level.

**Conclusion:**

Trachoma has not been eliminated from Car-Nicobar Island in accordance to ‘Global Elimination of Trachoma, 2020’ guidelines. Sustained efforts and continuous surveillance admixed with adequate programmatic response is imperative for elimination of trachoma in the island.

## Introduction

Trachoma is one of the neglected tropical diseases targeted for global elimination by the year 2020 [1]. The impact of the disease can be exemplified by the economic burden that has been estimated to be around US$ 3 to 8 billion in terms of lost productivity [2]. Currently, 53 countries globally are believed to be endemic for trachoma [3]. The number of people living in the trachoma endemic districts has been estimated to have reduced from 314 million in 2011 to 229 million in 2013 [4]. The World Health Organization (WHO) endorsed the SAFE (Surgery, Antibiotics, Facial cleanliness, Environmental modification) strategy in 1997, that has been implemented in different countries and has yielded significant gains in terms of reduction in the overall burden of trachoma in endemic regions.

India has come a long way from being trachoma-hyperendemic in 1950s, consequent to which, the trachoma control programme was initiated in 1963 [5]. Subsequently, trachoma was integrated into the National Programme for Control of Blindness (NPCB), India that was launched in 1976 [6]. The national blindness survey, India conducted during 1986–89 demonstrated significant reduction in trachoma-related blindness [7]. Concerted efforts are underway in the country to eliminate trachoma-related blindness as part of Global Elimination of Trachoma (GET) and Vision 2020 initiatives. The last NPCB-funded rapid assessment of trachoma (TRA) survey was undertaken in 2006–07 that reported the magnitude of active trachoma infection to be 5.8% (range 0.6 to 11.4%) in children aged 1–9 years and 0.1% (range 0.03 to 0.5%) trichiasis among adults [8]. This TRA however, was limited in its scope as it was undertaken in only six districts from northern and western parts of India that were earlier reported to be hyper-endemic. The NPCB, India has enforced routine reporting of trachoma cases from all parts of the country and population-based surveys are carried out in geographical regions that report high number of trachoma cases.

In the year 2010, an alarmingly high number of cases of active trachoma infection and trachomatous trichiasis were reported from Car-Nicobar Island, part of southernmost district of India that prompted NPCB, India to investigate the actual situation of trachoma in the island. The TRA conducted in the year 2010 revealed 50.5% children aged 1–9 years with evidence of active trachoma infection. The proportion of trichiasis amongst the participants aged 15 years and above, was reported to be 7.5% (range from 1% to 14.3% in different clusters). The burden was explained by unsatisfactory environmental conditions, rampant overcrowding and co-habitance of the tribal population with domestic animals [9]. Following the TRA, three rounds of Mass Drug Administration (MDA) with azithromycin were implemented in all the villages of this island by local health authorities involving village personnel from years 2010 to 2012. Additionally, health education messages were disseminated primarily to promote facial cleaniliness and hygeine in the villages. After three years of trachoma control activities in the island, NPCB, India undertook a detailed trachoma prevalence survey in the year 2013 to ascertain the prevalence of active trachoma infection and trachomatous trichiasis in Car-Nicobar Island. We report herein, the survey findings of current prevalence of trachoma, its risk factors and coverage of the Nicobari population with mass azithromycin treatment for control of trachoma.

## Materials and Methods

### Study Setting

Nicobar district is one of the three districts of Andaman and Nicobar Islands, the southermost Union Territory of India. The district has a population of 36,819 as per Census of India, 2011 [10]. There are 12 inhabited islands within the district that are divided into three developmental blocks (tehsils), namely Car-Nicobar, Nancowry and Campbell Bay. ‘Nicobarese’ is a generic name given to all indigenous people inhabiting the Nicobar group of islands.

The Car-Nicobar Island contributes to the maximum population of the entire district (population of 20,292) and comprises of 15 villages [11]. This island is situated in the south-east part of Bay of Bengal between 6 degree to 10 degree N latitude and 92 degree to 94 degree E longitudes. The climate of Car-Nicobar Island is tropical with an annual rainfall of 400 mm. It is a tribal reserve area and only restricted movement of people occurs to the mainland through limited transport channels like ship and helicopter. There have been no significant changes in terms of migration of islanders since the last trachoma assessment survey (2010). In terms of health care facilities, the island has one major secondary level hospital and 11 sub-centres that constitute the primary health care system of the island. At the time of the present survey, no ophthalmologist was posted in the island and eye care services are rendered through an ophthalmic technician. Surgical management for trichiasis and other ocular diseases is available through the referral hospital at Port Blair, 143 miles distant from this island and separated by sea.

### Sampling procedure and sample population

This was a population-based cross sectional prevalence survey that was conducted in all the fifteen villages of Car- Nicobar island. For sample size estimation, the assumed prevalence of active trachoma infection was taken as 5% amongst children aged 1–9 years. This assumption was made considering trachoma elimination has been achieved in most of the villages of the island after three rounds of MDA. On taking 2.5% absolute precision, design effect of 2.5 and 5% non-response rate, a minimum sample of 798 children was calculated for this survey. Compact segment cluster sampling technique was employed for this survey. Each village was divided into compact segments comprising of 175–350 people of all age groups. It was envisaged that each compact segment would at least have 50 children aged 1–9 years for assessment of active trachoma infection. In each village, one segment was randomly chosen by lottery method using concealed envelopes. The local village head (captain) was requested to pick up one number inside the concealed envelopes. All households were covered in the selected segment. The children aged 1–9 years were examined for signs of active trachoma infection and participants aged 10 years and above were examined for presence of trachomatous trichiasis and its sequale. Infants less than one year of age were not included in the study.

### Training of team members and quality assurance

The survey teams comprised of epidemiologists, ophthalmologists, ophthalmic assistants (OA), field supervisors, field workers and volunteers. A two-day training programme was conducted for all team personnel. The team members were trained by senior investigators about data collection procedures, filling up of relevant household and participant information and preparation of conjunctival smears. The ophthalmologists, involved in the trachoma survey, possessed adequate training in the cornea and anterior segment sub-specialty at the apex tertiary eye care institute of India and had extensive experience in identifying, diagnosing and managing trachoma in children and adults. The participating ophthalmologists attended training sessions based on identification of stages of trachoma as per WHO simplified grading system [12]. They were then assessed for their agreement with the standard WHO trachoma slides, considered gold standard for ascertaining agreement. The kappa coefficient was more than 0.8, indicative of good agreement. One of the ophthalmologists in the survey team (NG), with more than ten years of experience in treating and diagnosing corneal diseases, especially trachoma, had participated in the rapid assessment survey in Car-Nicobar Island during 2010 and was hence, familiar with the local ocular conditions and morbidity pattern. Additionally, a senior local ophthalmologist (AS) from Port Blair, Andaman & Nicobar Islands, India was part of the supervisory team of the present survey. With more than twenty years of experience in comprehensive eye care including trachoma, this ophthalmologist was involved in the previous TRA survey, 2010 was well versed with local ocular morbidity profile.

A pilot testing of survey procedures was done before the start of the survey. The two day pilot exercise was undertaken separately in an urban slum of Delhi and in Port Blair. Survey procedures including form filling, examination procedures, facial cleanliness status, trachoma grading and conjunctival smear preparation were standardized so as to assure quality data collection. The field work was supervised in each segment by a senior investigator (PV, SM & AS). The data was collected using pre-coded questionnaires.

### Ethics statement

The survey was conducted in accordance to the principles guided through the Declaration of Helsinki and ethical clearance for this survey was taken from the Institute Ethics Committee, All India Institute of Medical Sciences (AIIMS), New Delhi. Local approval was taken from tribal council, administrative authorities and village heads. Written informed consent was obtained from a responsible adult member of the household before acquiring any information and examination of the household members. Parental consent was sought before obtaining conjunctival samples of children with active trachoma infection. These children were treated with oral azithromycin after preparation of conjunctival smears at their respective primary health centres. The adults with trichiasis or corneal opacity due to trachoma were referred to Port Blair for appropriate management. Data was analyzed in aggregate maintaining confidentiality of survey participants.

### Data collection and clinical assessment

All the village heads were sensitized in advance and informed about the survey procedures. The survey was conducted during March-April 2013. From each household, demographic and clinical data was collected from each family member and operational definitions related to the family and environmental factors are presented below:

Family member-Individuals living together under the same roof and sharing a common kitchen, where food is cooked for the entire family.
Education- was enquired as completed years of education. It was dichotomized into primary level (those who could read and write and/or were educated upto fifth standard of formal schooling) and those educated above primary level.
Water source distance- Walking distance of water source from the household whether more than 30 minutes or not.
Presence of latrine-Sanitary latrine of any type within the household premises and being used routinely by the family members.
Presence of solid waste within 20 meters of household [13]- Solid waste included garbage (food wastes), rubbish, demolition products, sewage treatment residue, faecal waste from animals, dead animals, manure and other discarded material.
Presence of animals within 20 meters of household- All animals co-habiting the household as pets or were found near the household were included like dogs, cats, hens, pigs etc.
Overcrowding: This was expressed as the number of persons per room- number of persons living in the household divided by the total number of living rooms in the house, excluding the kitchen and bathroom. More than 2 persons per room was considered as overcrowding [14,15].

Coverage with azithromycin during the three subsequent years (2010 to 2012) was also ascertained for all enumerated individuals. The history regarding receipt of azithromycin for children was primarily obtained from mother or seniormost available member of the household. Infants, pregnant and lactating women were not given azithromycin during MDA and infants for their first year, born in the last three years were excluded for computing coverage. This was verified with written, documented records with NPCB, Port Blair where the data was maintained for all the villages across all the three years.

Ophthalmic examination was performed using torch light and a 2.5x binocular loupe. The WHO simplified grading system was used to determine the stage of clinical trachoma in survey participants. Children aged 1–9 years were examined for active trachoma infection. This was considered to be present when either features suggestive of trachomatous inflammation- Follicular (TF) and/or trachomatous inflammation-Intense (TI) were detected in either eye. TF was defined as presence of five or more follicles (at least 0.5 mm in size) in the upper tarsal conjunctiva. TI was considered when there was pronounced inflammatory thickening of the upper tarsal conjunctiva obscuring more than half of the deep tarsal vasculature of the upper lid. The surveyed children were also examined for presence of unclean face utilizing the definition used by earlier studies (with any one of the following features—nasal or ocular discharge or flies around nose/ eyes) [16]. The face of the child was examined first followed by eyes.

Participants aged ten years and above were examined for signs of trachomatous trichiasis (TT) and corneal opacity (CO) in either eye. For labeling TT, evidence of at least one eyelash rubbing the eye ball was looked for and/or recent history of removal of any inturned eyelash was considered as TT. Recurrent TT was defined as re-appearance of trichiatic eyelash or in-turned eyelid due to trachoma even after undergoing trichiasis or entropion surgery. The corneal opacity (CO) was labeled to be trachomatous when co-existing signs of trachomatous infection and signs of healed infection in form of conjunctival scarring (Arlt’s line) and trichiasis were present. The other etiologies of CO like vitamin A deficiency, trauma, keratitis, ocular surface disease, corneal degeneration etc. were ruled out by the ophthalmologist. Presenting visual acuity was measured in both eyes using Snellens E chart in patients detected with corneal opacity in any eye. In the present study, blindness was defined as presenting visual acuity of less than 3/60 in the better eye.

### Microbiological investigations

Conjunctival swabs were taken by the ophthalmologists from all the children found to be positive for clinical trachoma after observing standard universal precautions, mainly to corroborate the clinical findings. On completion of the compact segment cluster in each village, all children detected with trachoma infection on clinical examination, were brought to a nearby health centre for sample collection. After thorough swabbing and rolling of the entire upper conjunctival surface by a sterile swab stick, a smear was prepared on a clean teflon-coated glass slide marked with a unique identification number. All the slide specimens were air dried, fixed in acetone for ten minutes and stored in an ice box for maintaining the cold chain. The samples were transported from the villages to the Bishop John Hospital, Car Nicobar, which was at a distance of 1–10 km from all surveyed villages. At the hospital, the samples were stored and frozen in the refrigerator (at a temperature of zero degree celsius). On completion of the trachoma prevalence survey at Car-Nicobar island, samples were transferred in an ice box by ship and air with the survey team to Ocular Microbiology department at Dr. Rajendra Prasad Centre for Ophthalmic Sciences, AIIMS, New Delhi for further analysis.

Direct Immunofluorescence assay was performed using the MicroTrak Chlamydia *trachomatis* Direct Specimen Kit procured from M/s Trinity Biotech, Ireland^®^ for detection of *Chlamydia* antigen using the standard protocol [17]. A positive control and a negative control, as provided by the supplier, were processed along with each set of specimens to ensure reliability of the reagents. Morphology for positive specimens was confirmed at a magnification of 1000x. All slides were screened for a minimum of 100 high power fields. Specimens were considered positive only if a minimum of 10 smooth elementary bodies (apple green, regular, refractile and fluorescent indicative of Chlamydia *trachomatis*) were observed [18].

### Data entry and statistical analysis

Data entry was performed using Microsoft Access and double data entry was also done to catch and correct random miskeyed strokes. Statistical analysis was carried out using Stata 12.0 (Stata Corp., College Station, TX, USA). Data were presented as number (percentage). Village level segregated percentages are reported keeping in mind the variations that might happen for undertaking trachoma elimination efforts in the villages. The risk factors, (individual, household and village level) associated with active trachoma infection and trichiasis were analyzed using logistic regression for survey analysis (*svy*:*logit* command). All the risk factor variables were included in the multivariate analysis irrespective of its significance level, owing to limited factors and keeping in mind that all were equally important factors. Each of the outcomes were analyzed twice, i.e., in model-1 household as cluster and in model-2 village as cluster. The results were presented as odds ratio (95% C.I.). The p-value less than 0.05 was considered as statistically significant.

## Results

A total of 4178 participants (range of 182 to 382 per village) were enumerated in 15 villages of Car-Nicobar Island as shown in Table 1. Out of these, 847 were aged 1–9 years and amongst them, 809 (95.5%) were examined. The mean (SD) age of children examined was 5 (±2) years and was similar for boys and girls. A total of 2735 persons aged 10 years and above (82% response rate) were examined for signs of trachoma. The mean (SD) age for males examined in this group was 40 (±19) years and for females was 37 (±17) years.

**Table 1:** Profile and coverage of study population for the trachoma prevalence survey in the 15 village clusters of Car-Nicobar Island.

Villages	Total population enumerated N	Total population examined n (%)	Children aged 1–9 years enumerated n	Children aged 1–9 years examined n (%)	People aged ≥ 10 years enumerated N	People aged ≥ 10 years examined n (%)
Kinyuka	350	291 (83.1)	56	53 (94.6)	294	238 (81.0)
Perka	221	186 (84.2)	56	54 (96.4)	165	132 (80.0)
Tamalu	273	226 (82.8)	60	58 (96.7)	213	168 (78.9)
Malacca	382	322 (84.3)	58	54 (93.1)	324	268 (82.7)
Small Lapathy	207	191 (92.3)	53	52 (98.1)	154	139 (90.3)
Kinmai	256	234 (91.4)	52	50 (96.2)	204	184 (90.2)
Mus	367	327 (89.1)	58	57 (98.3)	309	270 (87.4)
Teetop	258	233 (90.3)	51	51 (100)	207	182 (87.9)
Sawai	257	205 (79.8)	57	54 (94.7)	200	151 (76.1)
Big Lapathy	251	202 (80.5)	50	49 (98.0)	201	153 (76.1)
Kakana	250	197 (78.8)	58	54 (93.1)	192	143 (74.5)
Kimious	242	199 (82.2)	53	52 (98.1)	189	147 (77.8)
Arong	182	136 (74.7)	59	54 (91.5)	123	82 (66.7)
Tapoiming	317	304 (95.9)	60	57 (95.0)	257	247 (96.1)
Chukchuka	365	291 (79.7)	66	60 (90.9)	299	231 (77.3)
**Total**	**4178**	**3544 (84.8)**	**847**	**809 (95.5)**	**3331**	**2735 (82.1)**

### Household characteristics

The enumerated participants were enrolled from a total of 552 households (range of 26 to 56 households per village). The household characteristics are represented in Table 2. Majority of the households had three risk factors conducive for transmission of trachoma in the community. Overcrowding was observed in 76% of households. Solid waste within 20m of household was observed in around 90% of the surveyed households. Predominantly, all the households (98%) had animals (like hens, pigs, dogs, cats etc.) in their vicinity. Presence of functional latrine and availability of water source within a walking distance of 30 minutes was found in 99% of households.

**Table 2:** Household characteristics and distribution of risk factors for Trachoma in Car-Nicobar Island.

Villages	Number of Household n	Overcrowding Present n (%)	Water Source >30 min of walking distance n (%)	Presence of Solid waste near household n (%)	Absence of latrine n (%)	Animals around household n (%)
Kinyuka	56	34 (60.7)	1 (1.8)	49 (87.5)	0 (0.0)	55 (98.2)
Perka	35	32 (91.4)	0 (0.0)	31 (88.6)	0 (0.0)	35 (100.0)
Tamalu	37	27 (73.0)	0 (0.0)	35 (94.6)	0 (0.0)	36 (97.3)
Malacca	46	31 (67.4)	4 (8.7)	46 (100.0)	1 (2.2)	44 (95.7)
Small Lapathy	21	19 (90.5)	0 (0.0)	15 (71.4)	0 (0.0)	21 (100.0)
Kinmai	35	29 (82.9)	0 (0.0)	35 (100.0)	0 (0.0)	35 (100.0)
Mus	43	32 (74.4)	0 (0.0)	42 (97.7)	1 (2.3)	39 (90.7)
Teetop	31	26 (83.9)	1 (3.2)	22 (71.0)	0 (0.0)	31 (100.0)
Sawai	33	28 (84.8)	0 (0.0)	31 (93.9)	1 (3.0)	31 (93.9)
Big Lapathy	38	25 (65.8)	0 (0.0)	38 (100.0)	0 (0.0)	38 (100.0)
Kakana	41	35 (85.4)	0 (0.0)	41 (100.0)	0 (0.0)	41 (100.0)
Kimious	39	34 (87.2)	0 (0.0)	24 (61.5)	0 (0.0)	39 (100.0)
Arong	26	18 (69.2)	0 (0.0)	26 (100.0)	0 (0.0)	26 (100.0)
Tapoiming	32	28 (87.5)	0 (0.0)	32 (100.0)	0 (0.0)	32 (100.0)
Chukchuka	39	22 (56.4)	1 (2.6)	28 (71.8)	1 (2.6)	39 (100.0)
**Total**	**552**	**420 (76.1)**	**7 (1.3)**	**495 (89.7)**	**4 (0.7)**	**542 (98.2)**

### Coverage for Mass Drug Administration (MDA) for trachoma in Car Nicobar

The coverage with oral azithromycin for three consecutive years in the examined population was found to be 90.2% for 2012, 87.6% for 2011 and 83.2% for 2010 respectively.

### Prevalence of active trachoma infection in Car-Nicobar

A total of 55 cases with active trachoma infection were identified with a prevalence of 6.8% (95% CI 5.1, 8.5) as shown in Table 3. The prevalence of active trachoma infection was more than 10% in three villages namely in Small Lapathy (23%), Perka (22%) and Teetop (12%). Cases with active trachoma infection were seen in 12 villages except in Malacca, Mus and Big Lapathy. All the cases demonstrated follicular stage of trachoma, while there was no case of trachomatous inflammation (TI) in the study population. Conjunctival swab specimens could be collected from the upper conjunctival surface of 46 children with active trachoma infection. Out of these, 39 samples were found positive (84.8%) by the direct immunofluorescent assay for *Chlamydia trachomatis*.

**Table 3:** Distribution of Trachoma Cases in Car-Nicobar Island.

Villages	Total population	Children aged 1–9 years examined n	Children with Active Trachoma n (%)	People aged ≥ 10 years examined n	People aged ≥ 10 years with trachomatous trichiasis n	Prevalence of TT/1000 people aged ≥ 10 years	Prevalence of TT/ 1000 population
Kinyuka	1120	53	04 (7.5)	238	09	38	30
Perka	2527	54	12 (22.2)	132	03	23	18
Tamalu	1515	58	03 (5.2)	168	14	83	67
Malacca	1637	54	00 (0.0)	268	12	45	36
Small Lapathy	938	52	12 (23.1)	139	01	07	06
Kinmai	574	50	02 (4.0)	184	07	38	30
Mus	1553	57	00 (0.0)	270	13	48	39
Teetop	522	51	06 (11.8)	182	05	27	22
Sawai	1247	54	01 (1.9)	151	02	13	11
Big Lapathy	1098	49	00 (0.0)	153	05	33	26
Kakana	841	54	05 (9.3)	143	05	35	28
Kimious	382	52	04 (7.7)	147	01	07	05
Arong	1194	54	03 (5.6)	82	02	24	20
Tapoiming	941	57	01 (1.8)	247	19	77	62
Chukchuka	1021	60	02 (3.3)	231	09	39	31
**Total**	**17110**	**809**	**55 (6.8)**	**2735**	**107**	**39**	**30**

### Risk factors for presence of active trachoma infection (TF)

The risk factors associated with active trachoma infection in the sampled children are shown in Table 4. The results were similar in both the models when household and village were treated as clusters independently. With every increase in one year of age in children, the odds of having active trachoma infection in children increased by 20% [adjusted OR 1.20 (95% CI 1.08, 1.34)]. There was no sex predeliction for presence of TF. Unclean faces were observed in 5% of the total children examined. Amongst children with unclean faces, 21% had TF. The odds of having TF amongst children with unclean faces was found to be five times more than in children with clean faces [adjusted OR 4.90 (95% CI 2.17, 11.09); p <0.001].

**Table 4:** Association of Risk factors for active trachoma in children (1–9 years) of Car-Nicobar Island with Household and Village clustering effect.

Variables (n)	TF* positive children (%)	Odds Ratio (95% CI)			
		Model-1 (Household as cluster)		Model-2 (Village as cluster)	
		Unadjusted	Adjusted	Unadjusted	Adjusted
Age (809)	5.8 (2.1)**	1.2 (1.1, 1.3)	1.2 (1.08, 1.34)	1.17 (1.08, 1.27)	1.2 (1.09, 1.33)
Sex					
Male (424)	29 (6.8)	1.00	1.00	1.00	1.00
Female (385)	26 (6.8)	0.99 (0.57, 1.72)	1.00 (0.57, 1.78)	0.99 (0.64, 1.51)	1.00 (0.60, 1.67)
Unclean face					
Absent (767)	46 (6.0)	1.00	1.00	1.00	1.00
Present (42)	9 (21.4)	4.27 (1.99, 9.19)	4.58 (2.10, 10.00)	4.27 (1.53, 11.95)	4.58 (1.77, 11.83)
Solid waste near household					
Absent (107)	12 (11.2)	1.00	1.00	1.00	1.00
Present (702)	43 (6.1)	0.52 (0.25, 1.06)	0.49 (0.22, 1.08)	0.52 (0.30, 0.90)	0.49 (0.28, 0.85)
Overcrowding					
Absent (102)	7 (7.1)	1.00	1.00	1.00	1.00
Present (707)	48 (6.8)	0.99 (0.43, 2.27)	1.09 (0.47, 2.56)	0.99 (0.32, 3.02)	1.09 (0.38, 3.13)
Education of head of household					
Upto Primary Level (435)	27 (6.2)	1.00	1.00	1.00	1.00
Above Primary Level (374)	28 (7.5)	1.22 (0.73, 2.05)	1.21 (0.72, 2.04)	1.22 (0.79, 1.89)	1.21 (0.80, 1.84)
Water Source > 30 min of walking distance					
No (799)	54 (6.8)	1.00	1.00	1.00	1.00
Yes (10)	1 (10.0)	1.53 (0.21, 11.1)	2.01 (0.24, 16.96)	1.53 (0.10, 23.07)	2.00 (0.10, 38.49)

*-TF = Trachoma folliculare;—CI: Confidence Interval

**-age 5.8 (2.1) represents mean (SD)

### Prevalence of Trachomatous Trichiasis (TT) and Corneal Opacity (CO)

Cases with TT were identified in all the 15 village segments ranging from one case in Small Lapathy to 19 cases in Tapoiming. A total of 107 cases of TT were detected with an overall prevalence of 39 per 1000 persons aged 10 years and above (95% CI 31.8, 46.3). Corneal opacity was found in 27% (29) cases with trichiasis. The prevalence of CO due to trachoma was 1.1% in the examined population. Nine patients had bilateral CO due to TT. Three patients were blind due to trachomatous CO. Recurrent trichiasis was found in six (0.2%) cases. The interval between surgery and recurrence of TT varied in different individuals and it ranged from 3 months to 3 years.

### Risk factors for Trachomatous Trichiasis (TT)

The risk factors found to be associated with TT are shown in Table 5. The results were similar in both the models when household and village were treated as clusters independently. The occurrence of TT was positively correlated with age. The odds of having trichiasis increased by 5% for every year increase in age [adjusted OR 1.05 (95% CI 1.04, 1.06)]. There was no significant difference between males and females with respect to occurrence of TT. TT was seen more frequently (8%) in persons who were less literate (upto primary level education level) as compared to those who had received higher education (2%). The odds of having TT was 58% lower in people with higher level of education as compared to lesser educated population [adjusted OR 0.42 (95% CI 0.26, 0.67)].

**Table 5:** Association of Risk factors of Trachomatous Trichiasis (TT) in population ≥ 10 years of Car-Nicobar Island with Household and Village clustering effect.

Variables (n)	TT* positive adults (%)	Odds Ratio (95% CI)			
		Model-1 (Household as cluster)		Model-2 (Village as cluster)	
		Unadjusted	Adjusted	Unadjusted	Adjusted
Age (2735)	57.4 (13.2)**	1.06 (1.05, 1.07)	1.05 (1.04, 1.06)	1.06 (1.05, 1.07)	1.05 (1.04, 1.06)
Sex					
Male (1138)	49 (4.3)	1.00	1.00	1.00	1.00
Female (1597)	58 (3.6)	0.84 (0.57, 1.23)	0.91 (0.60, 1.37)	0.84 (0.59, 1.19)	0.91 (0.59, 1.39)
Education					
Upto Primary Level (973)	79 (8.1)	1.00	1.00	1.00	1.00
Above Primary Level (1762)	28 (1.6)	0.18 (0.12, 0.27)	0.42 (0.26, 0.69)	0.18 (0.11, 0.30)	0.42 (0.25, 0.73)
Overcrowding					
Absent (479)	25 (5.2)	1.00	1.00	1.00	1.00
Present (2247)	82 (3.6)	0.72 (0.44, 1.18)	0.75 (0.44, 1.26)	0.72 (0.46, 1.11)	0.75 (0.42, 1.32)
Water Source > 30 min of walking distance					
No (2689)	106 (3.9)	1.00	1.00	1.00	1.00
Yes (46)	1 (2.2)	0.54 (0.09, 3.17)	0.56 (0.08, 4.05)	0.54 (0.20, 1.47)	0.56 (0.18, 1.78)
Animals around household					
Absent (45)	3 (6.7)	1.00	1.00	1.00	1.00
Present (2690)	104 (3.9)	0.56 (0.24, 0.33)	0.60 (0.22, 1.69)	0.56 (0.26, 1.21)	0.60 (0.29, 1.23)
Heard about Trachoma					
No (1146)	47 (4.1)	1.00	1.00	1.00	1.00
Yes (1589)	60 (3.8)	0.92 (0.61, 1.37)	0.87 (0.57, 1.32)	0.92 (0.50, 1.68)	0.88 (0.48, 1.58

*-TT = Trachomatous Trichiasis; —CI: Confidence Interval

**-age 57.4 (13.2) represents mean (SD)

## Discussion

The present study involved a cross sectional prevalence survey to ascertain the status of trachoma infection and its sequelae in the 15 villages of Car-Nicobar Island, Andaman & Nicobar Islands, India. After three rounds of MDA with azithromycin from 2010–2012, the prevalence of trachoma follicular stage in children was found to be 6.8% (95% CI 5.1, 8.5) and that of TT in adults was 3.9% (95% CI 3.18, 4.63). Based on these findings, it could be inferred that trachoma had not been eliminated from the villages of this underserved, tribal reserve area.

The prevalence of active trachoma infection in seven villages was below 5% and this indirectly highlighted the positive effect of MDA in these villages. Five of the villages surveyed had prevalence of active trachoma infection between 5–10% and another round of MDA in these villages might be helpful in lowering the prevalence of TF below 5%. However, three villages reported a high prevalence of active trachoma infection ranging from 12 to 23% and reflected the need for continuing with more MDA rounds in these villages. A high proportion (85%) of clinically positive trachoma cases was confirmed to be *Chlamydia trachomatis* by direct immunofluroscence assay. In the current study, older children demonstrated a higher prevalence of active trachoma infection. This is in contrast to earlier reports wherein higher prevalence of TF is seen in younger age group [19,20]. This could be explained that the island children who were older had been infected prior to MDA initiation and their infection was persisting. The younger cohorts might be offered a better protection from new infections owing to reduced circulation of C.*trachomatis* due to continuous rounds of MDA and improvements in environmental conditions and hygienic measures undertaken by villagers.

In our study, unclean faces amongst children came out to be an independent significant risk factor for active trachoma infection. Dirty faces, consistently was seen to enhance the risk of TF, as has been reported through other studies conducted in trachoma endemic communities [19,20,21]. There was a need for augmenting the behavior change messages to the communities for cleaning their face regularly. Other important factors favorable for trachoma transmission [22,23,24,25]in villages of Car-Nicobar, were presence of animals, solid waste and overcrowding in surveyed households.

High prevalence (30 per 1000 population) of TT was reported in the adult population of Car-Nicobar Island. Significant risk factors for presence of TT were higher age group and lower educational status. These findings are consistent with risk factors reported in other endemic communities [26]. In an Ethiopian community, adults aged more than 40 years, had twice the risk of having TT and and illiterates reported three times higher odds of trichiasis [27]. The current trichiasis load in the surveyed villages, pointed out the need for surgical facilities for managing trichiasis, entropion and corneal blindness in the island. Car-Nicobar Island being remotely situated, suffered geographic challenges. None of the villages had an access to trichiasis surgical management facility within the island. For any surgical intervention related to eye care; patients needed to be transferred to Port Blair, which was connected to this island by ship or helicopter services only.

Our study had some limitations. First, we did not calculate the sample size separately for estimating TT, all household members in the selelcted compact segment were included in the study. Similar practice of choosing adult members from the same households had also been reported in the Global Trachoma Mapping Project, undertaken in 34 countries [28]. We included adults aged 10 years and above for clinical examination of TT instead of standard practice of inclusion of adults aged 15 years and above due to operational difficulty in excluding 10–15 year members from clinical examination, considering the population belonging to tribal reserve areas of India. It is also worthwhile to mention that population based surveys for trachoma are challengining and are affected by sample size considerations and resources. This is evident by varying methodologies adopted in different settings where population based surveys related to trachoma have been undertaken and published, owing to non availability of a standard prescriptive methodology for same [28,29,30]. This was the first prevalence study undertaken for trachoma in a difficult to reach tribal population in an Indian island setting and would be imperative for future comparisons and evaluating trachoma elimination efforts in this area. Second, we attempted microbiological investigation for confirming *chlamydia trachomatis* but did not test children who were negative for active trachoma infection. The prime reason for testing children was to test feasibility of this approach in a difficult Indian setting. Operationally, it was difficult for us to take consent from this tribal population for testing children who did not have active infection infection. We used rapid antigen detection by immunofluorescence assay as it was relatively quicker and logistically easier to perform than PCR (Polymerase Chain Reaction) assay as samples were collected in Car Nicobar Island and had to be transported to Delhi, for processing. It was also more affordable than PCR assay and was time tested in other epidemiological studies from developing nations [31,32,33]. Third, our definitions utilized for testing environmental factors were obtained from varying sources and these could vary from one survey to another. However, studying these factors was important, so as to stress on modifiable behavior change in these communities and institutionalize SAFE interventions in these villages. Fourth, the data related to MDA coverage was subject to recall bias as it was collected primarily based on history through adult respondents.

Trachoma control efforts would need to be sustained further in villages of Car-Nicobar for complete elimination of trachoma. Local health workforce needs to be trained and to pick up cases with active trachoma infection along with its sequelae and manage them appropriately to halt further transmission and prevent needless blindness due to trachoma. Efforts need to be enhanced further to change behavior regarding facial cleanliness in children. A systematic review of randomized trials done in trachoma-endemic countries, provided some evidence that face washing in combination of antibiotics could be helpful in reducing severe active trachoma infection and increasing the prevalence of clean faces [34] School and community-based interventions should be scaled up further, to promote face washing in this setting.

The present effort of trachoma control programme in Car-Nicobar Island reflected the commitment of NPCB, Government of India in eliminating trachoma from the nation. Future efforts need to be continued in the island so as to achieve trachoma elimination. Concerted synergistic action from WASH (Water, Sanitation and Hygiene) departments locally would accelerate the progress towards achieving elimination. Further trachoma prevalence surveys in this area are recommended keeping in mind the methodological limitations of the current study and evaluating the future programmatic response for trachoma elimination.

## Supporting Information

S1 File Report. NPCB Trachoma Survey 2013 in Car-Nicobar Island, India.
(PDF)
