# Supplementary material for: Prevalence of Trachoma in Car-Nicobar Island, India after Three Annual Rounds of Mass Drug Administration with Azithromycin
Source: PLoS One. 2016 Jul 8;11(7):e0158625. doi: 10.1371/journal.pone.0158625 (PMC4938255; doi:10.1371/journal.pone.0158625)
Supplement: S1 File — NPCB Trachoma Survey 2013 in Car-Nicobar Island, India. (PDF) [file pone.0158625.s001.pdf]

NPCB Trachoma Survey 2013
in
Andaman & Nicobar Island, India

National Programme for Control of Blindness Ministry of Health & Family Welfare Government of India

NPCB Trachoma Survey 2013 in

Andaman & Nicobar Island, India

A REPORT

Coordinated by:
Community Ophthalmology Department,
Dr. Rajendra Prasad Centre for Ophthalmic Sciences,
AIIMS, New Delhi

MARCH- APRIL 2013

REPORT WRITING

Dr. Praveen Vashist

Dr. Sumit Malhotra

Dr. Noopur Gupta

Dr. R. P. Centre for Ophthalmic Sciences, AIIMS, New Delhi

National Program for Control of Blindness in India,
Directorate General of Health Services,
Ministry of Health & Family Welfare,
Government of India, New Delhi

EXPERT ADVISORY COMMITTEE:

1. Ms. Sujaya Krishnan, Joint Secretary, MOH&FW, Govt of India, Nirman Bhawan, New Delhi.
2. Dr. N. K. Agarwal, Deputy Director General (O), DGHS, MoH&FW, Govt of India, Nirman Bhawan, New Delhi.
3. Prof. Rajvardhan Azad, Chief, Dr. R. P. Centre, AIIMS, New Delhi.
4. Dr. Praveen Vashist, Additional Professor of Community Ophthalmology, Dr. R.P. Centre, AIIMS, New Delhi.
5. Dr. Sumit Malhotra, Assistant Professor of Community Ophthalmology, Dr. R.P. Centre, AIIMS, New Delhi.
6. Dr. Anita Shah, Deputy Director (Ophthalmology), State Ophthalmic Cell, Port Blair
7. Dr. Noopur Gupta, Scientist Grade III (Ophthalmologist), Dr. R.P. Centre, AIIMS, New Delhi.

KEY INVESTIGATORS/ COLLABORATORS

Dr. R. P. Centre for Ophthalmic Sciences, AIIMS, New Delhi

1. Prof. Rajvardhan Azad, Chief, R.P.Centre
2. Dr. Praveen Vashist- Additional Professor
3. Dr. Sumit Malhotra-Assistant Professor
4. Dr. Noopur Gupta- Scientist Grade III (Ophthalmologist)
5. Dr. Saurabh Agarwal- Ophthalmologist
6. Dr. Babulal- Ophthalmologist

G. B. Pant Hospital, Port Blair, Andaman & Nicobar Islands

1. Dr. Anita Shah-Deputy Director (Ophth), State Ophthalmic Cell, Port Blair.

BJR Hospital, Car Nicobar, Island

1. Dr. B. G. Lal-Medical Superintendent, BJR Hospital, Car Nicobar

Special Thanks for the administrative and technical support provided during the survey:

1. Shri P. Jawahar, IAS, District Commissioner, Nicobar District.
2. Prof. Geeta Satpathy & Prof. N. Nayak, Department of Ocular Microbiology, Dr. R.P. Centre, AIIMS, New Delhi.
3. Ophthalmic technicians, supervisors, LHV, ASHAs & ANMs involved in survey.
4. Village Captains of all the villages in Car Nicobar.

Preface

Trachoma is a priority eye condition, responsible for maximum blind persons attributable to infectious blindness worldwide. The disease is still endemic in certain countries globally. Many nations are in process of getting verification for trachoma elimination.

India is committed to achieve trachoma elimination as a signatory to global elimination of trachoma by year 2020. Trachoma control efforts are part of National Programme for Control of Blindness implemented all over the country. Trachoma related blindness was a major public health problem in India during 1959-63 with hyperendemic trachoma rates for active infection. Since then, public health efforts were institutionalized to reach out to masses with basket of trachoma interventions- SAFE (Surgery, Antibiotics, Facial cleanliness and Environmental Modifications). The last multi-district rapid assessment for trachoma was conducted in India during 2006, reported around 6% of children to have active trachoma and very low magnitude of trichiasis (0.2%).

As part of regular monitoring from states and districts, trachoma cases are reported and if found in excess, epidemiological investigations are conducted in different parts of the country. Dr. Rajendra Prasad Centre for Ophthalmic Sciences, as a technical body provides inputs to National Programme for Control of Blindness including trachoma control activities. The Community Ophthalmology department at Dr. RP Centre assists in undertaking several epidemiological assessments. Nicobar Islands was hyper-endemic for trachoma where a situation analysis was conducted during 2010. The present report captures the current trachoma situation in Nicobar Islands and selected clusters of Andaman Union Territory. I am hopeful that through surveillance efforts and SAFE interventions, India will be able to eliminate trachoma in entire country.

Prof. Rajvardhan Azad

INDEX

Summary at a glance

Survey Photographs

Trachoma Prevalence Survey in Nicobar

Background

Objectives

Methodology

Results

Conclusion & Recommendations

Rapid Assessment Trachoma Survey in Andaman

Abbreviations

Annexures

NPCB Trachoma Survey 2013 in Andaman & Nicobar Island, India

Summary at a glance

Indicators	Results
**Trachoma Prevalence Survey in Car Nicobar island**	
Prevalence of active trachoma infection (TF+TI)0 in children aged 1-9 years	6.8% (95% CI 5.1 - 8.5)
Prevalence of unclean faces	5.2 %
Microbiological test positivity for trachoma amongst samples tested	84.8%
Prevalence of trichiasis (TT) in poplulation 10 years and above	3.9%
Coverage of mass drug administration in 2012, 2011 and 2010	90.2%, 87.6% and 83.2% respectively
Reduction in active trachoma infection	50.8% (2010) to 6.8% (2013)

Trachoma Prevalence Survey in Car Nicobar Island, India

1. Background

Trachoma, the leading cause of infectious blindness globally, usually affects the most socio-economically disadvantaged regions of the world. According to recent estimates, trachoma is endemic in 57 countries of the world and India is one of the five countries accounting for nearly half of the global burden of active trachoma. Trachoma related blindness was a major public health problem in India in the mid-twentieth century with active trachoma rates as high as 79% in children under 10 years of age in some northern states of the country. The last national survey on trachoma under National Programme for Control of Blindness (NPCB), India was conducted in six states in 2006. This survey reported that 5.8% of children aged 1-9 years had signs of active trachoma infection, while the magnitude of trichiasis was very low (0.15%). The survey demonstrated that trachoma has ceased to be a public health problem in India.

India is committed to elimination of trachoma related blindness by2020 as partner to the alliance for the Global Elimination of Trachoma (GET) launched by the World Health Organization in the year 1997. In order to achieve this goal, remote, poor and marginalized populations of the country with poor socio-developmental indicators where trachoma is likely to be endemic, need to be surveyed for prioritizing interventions to eliminate trachoma.

Car-Nicobar Island, a restricted tribal area, is among the largest islands in the Bay of Bengal and the southernmost district of the country. NPCB conducted Rapid Assessment of Trachoma (TRA) survey in ten villages of Car Nicobar Island in 2010 according to standard WHO guidelines. The survey in Car Nicobar showed a very high active trachoma infection magnitude (TF/TI) of 50.8 % among children in 1-9 year age group ranging from 37.5 % to 73% in the ten village clusters included in the survey. Trachomatous trichiasis was noted in 7.5% of the population examined aged 15 years and above ranging from 1% to 14.3% in different clusters. The environmental sanitation was not found to be satisfactory in the surveyed villages mainly due to the co-habitance of Nicobari people with domestic animals and overcrowding.

Considering the high magnitude of active trachoma infection in children and trichiasis among adults, special initiatives were taken by the Government of Andaman & Nicobar Islands, India to implement SAFE strategy measures in the island. Surgical facilities for patients with trichiasis were made available by G B Pant Hospital, Port Blair. Local health teams involving ANMs and

ASHA workers were trained in preventive measures for trachoma control as well as in identification of TT cases and their referral for surgery in the hospital.

Mass azithromycin treatment was recommended for all the residents of Car Nicobar Island for three consecutive years as a single annual dose either in the form of tablets or oral suspension. The Village Captains, ANMs and ASHA workers in each village were involved in providing azithromycin treatment under the guidance of District Programme Manager and local ophthalmologist and optometrist.

The State Programme Officer for National programme for Control of Blindness informed the Ministry of Health & Family Welfare, Government of India that the Mass azithromycin treatment was implemented for three consecutive years in 2010, 2011 and 2012 in the island with coverage of more than 80% population in each round. On request of the local health authorities of Andaman & Nicobar Island, this prevalence survey was planned to assess the current burden of trachoma in the Nicobar Island along with the evaluation of the SAFE strategy measures in the Island.

2. Objectives of the Survey

- To estimate the prevalence of active trachoma infection (TF & TI) in Car Nicobar Island
- To determine the prevalence of trachomatous trichiasis (TT) and trachomatous corneal opacity (CO) in this island.
- To assess the current status of socio- environmental risk factors for trachoma
- To ascertain the coverage achieved with three rounds of mass azithromycin treatment in Car-Nicobar Island.

3 Methodology

3.3.1. Study Area:

Car Nicobar Island is situated in the south-east part of Bay of Bengal between 6° to 10° N latitude and between 92° to 94° E longitudes. The climate of Car Nicobar Island is tropical with an annual rainfall of 400 mm. Car Nicobar is 143 miles from Port Blair, capital of Andaman & Nicobar Islands. Nicobar district forms the southern-most part of India and has two tehsils of Car Nicobar and Nancowrie covering 1841 square kilometers. All the islands have been declared as tribal reserve area under Andaman and Nicobar (Protection of Aboriginal tribes) Regulation Act 1956.

'Nicobarese' is a generic name of all indigenous people inhabiting in Nicobar group of islands. They are off shoots of mongoloid race and share many cultural and social traits. One of them is the Tu-het, the extended household, a very important social unit among the Nicobarese, which controls the socio-economic activities of its members. Politically, one headman (captain) who is democratically elected heads every village. Noticeably, this election is an internal affair of the community. It is conducted without any government involvement. All such village headmen constitute tribal council headed by the Chief Captain, one for each island. Direct administration of the island is performed by the Car Nicobar tehsil, a local administrative division of the Nicobar district.

3.3.2 Study Duration:

The trachoma survey was conducted in fifteen days period in March-April, 2013 including the training sessions (Annexure 1)

3.3.3 Study Population, Sample Population & Sampling:

The island of Car Nicobar has an estimated population size of approximately 20292 as per census 2011. There are a total of 15 villages in the island namely Mus, Kinmai, Small Lapathy, Big Lapathy, Tapoiming, Chukchucha, Kinyuka, Tamalu, Perka, Malacca, Kakana, Kimiuos, Arong, Sawai & Teetop. The estimated sample size for the survey was 4500 which is around 20% of the total island population. It was planned to cover all the villages of Car Nicobar is land for the prevalence study. In each village, a segment/cluster comprising of nearly 250-350 people of all ages was selected randomly. It was estimated that each cluster would approximately have at least 50 children aged 1-9 years for assessment of active trachoma infection (TF/TI) and another 200-300 people aged 10 years and above for assessing trachoma sequalae (TT & CO). The sampling technique employed was cluster random sampling approach as recommended by World Health Organization for conducting trachoma prevalence surveys.

3.3.4 Survey Instruments:

The study tool involved a semi-structured questionnaire and observation of environmental risk factors. (Annexure 2) The different components of the study tool were:

A. Identification data for the cluster and household

B. Semi structured interview for knowledge about trachoma

C. Observation of environmental risk factors

D. Examination details of household members of 10 years and above age for trachoma sequalae and mass drug administration coverage.

E. Examination details of children aged 1-9 years for active trachoma infection and Mass Drug Administration coverage

3.3.5 Study Teams:

Three teams were involved in conducting the survey at Car Nicobar island. Each team consisted of five members i.e. Ophthalmologist, Ophthalmic Assistant, Field Supervisor, Field Investigator and a Field attendant. Beside this, local volunteers mainly ASHA or Anganwadi workers from the villages were also involved in the survey. All the three teams worked simultaneously in different clusters covering one cluster each day. All the 15 clusters were covered in five days.

3.3.6 Training for the Trachoma Survey:

The team members of R.P.Centre were trained for two days including piloting of survey in a village. The local teams from the Bishop John Hospital were trained for one day. The training schedule for R.P.Centre team is enclosed. (Annexure 3)

A four member expert team comprising of Chief Investigator, Epidemiologist, Senior Ophthalmologist and Microbiologist from Dr. R. P. Centre, AIIMS was involved in training of all the survey teams. The team members were oriented about methodology and operational aspects related to population based surveys. The training schedule included lecture demonstration, grading of the WHO trachoma slide set by the ophthalmologists and optometrists, role play on how to interview key informants, orientation to the different formats for data collection tools, filling up the tally sheets and clinical examination. Specific duties were given to each participant of the survey team member. The ophthalmologists were trained on WHO Trachoma Grading Slide set. The agreement analysis among three ophthalmologists was conducted both with standard WHO slides and also in field conditions. In the field, same group of 25 children in 1-9 year age group and 25 people of age 10 years and above were examined by each ophthalmologist. The senior most ophthalmologist was assumed as gold standard for comparison of agreement among the ophthalmologists. The agreement was above 80% both for WHO slides as well as in the field conditions among all three ophthalmologists.

The ophthalmologists were trained by the microbiologist from RP Centre, AIIMS for obtaining the tarsal conjunctival swab for microbiological investigation for Chlamydia trachomatous antigen detection. Storage and transport of the slides with these samples was also discussed and the importance of maintenance of cold chain was emphasized.

3.3.7 Field Survey & Data Collection

All the village captains were informed about the survey schedule and date and time of survey in their villages. The local volunteers, ASHA and Anganwadi worker of the village were identified and trained. The team of epidemiologist and field supervisor visited the village in advance. Maps of the villages were prepared with the help of volunteers. The team took a round of the entire village and randomly selected a cluster of 250-350 people in the village. The Village captains were requested to inform all the people in that cluster to be available in their households on the day of survey in order to ensure a good coverage.

All the clusters were assessed for facilities like availability of primary health centre, trichiasis

surgical facility, village pharmacy, market & schools in terms of the distance of these facilities. In recording the distance to a facility like PHC/trichiasis facility, it was decided that distance to all facilities within the village would be recorded in walking time while for all facilities outside the village it would be recorded in time taken by public transport.

For identifying a market, respondents were queried about the distance to shops selling groceries, vegetables and other items for daily living. Similarly for a pharmacy, respondents were queried about facility where common medicines for fever, malaria, cough and cold etc. were sold.

Three members of the survey team involving field supervisor, field investigator and one volunteer enumerated the household members and took consent from the head of household or any other responsible member of the family. They interviewed one adult member randomly selected from the household for knowledge about prevention of trachoma and trachomatous blindness. The team also assessed the environmental status of all the households in the cluster. The environmental risk factors assessed were distance of the water source, presence of solid waste & animals around the household and absence of functional sanitary latrine in the house. All such information was recorded in the survey forms. In villages with scattered houses, the distance criteria of 20 metres as recommended by WHO was followed.

The second team involving Ophthalmologist, Optometrist, Field attendant and a local volunteer was involved in ocular examination of all the enumerated people and collected conjunctival swabs of children with active trachoma. They also collected information about the mass drug administration for last three years in the enumerated population.

All the available household members above one year of age were examined by the ophthalmologist for signs of active trachoma in children aged 1-9 years and for trichiasis and its complications in all household members aged 10 years and above. Ocular examination for ascertaining signs of trachoma was performed with the help of 2.5x binocular corneal loupe and torch light. The grading system recommended by the WHO (FISTO classification) was used as given below. In cases with corneal opacity, visual acuity was also recorded in both eyes.

WHO classification for Trachoma grading (FISTO)

Trachoma follicular: (TF)	Presence of five or more follicles at least 0.5 mm in the upper tarsal conjunctiva.
Trachoma Inflammaiton Intense (TI)	Pronounced inflammatory thickening of the upper tarsal conjunctiva that obscures more than 50% of the deep conjunctival vessels.
Trachomatous Scarring (TS)	Presence of scarring in the tarsal conjunctiva
Trachomatous Trichiasis (TT)	At least one eyelash rubbing on the eyeball. Evidence of recent removal of in turned eyelashes was also graded as trichiasis
Corneal Opacity (CO)	Easily visible corneal opacity over the pupil

Trachoma is defined as eliminated if the prevalence of active infection is less than 5% in children aged 1-9 years. Interventions are needed if the prevalence of active trachoma is more than 5% in the study population.

Observation of facial hygiene was done on all the children examined for active trachoma. Unclean faces were defined as presence of discharge from the eyes / nose or crusting of discharge or presence of flies on the discharge around eye or nose.

3.3.8 Microbiological Investigations

If the child was found positive for active trachoma infection, the identification details were noted and conjunctival scrapings were taken after seeking informed consent from the parents. These children visited the nearest primary health centre or sub centre for these investigations and subsequent treatment with azithromycin tablets and eye drops was provided to children.

Children with trachoma were sampled for trachoma separately to avoid cross-contamination. Equipment and surfaces were cleaned with disinfectant between the collections of samples. Contamination was also avoided by rubbing sterilium (disinfectant) over the palmar and dorsal surface of hands and allowing it to dry after examining each eye. In children detected with active trachoma infection on clinical examination, the upper tarsal conjunctiva of each eye was everted and swabbed with sterile cotton swabs. The conjunctiva was swabbed (four times) with one side of the swab to collect cells from the entire exposed surface. The swab was then turned over and the swabbing was repeated to cover the entire upper conjunctival surface. A smear was made on a clean glass slide which was marked with the unique identification number of the child.

Direct Immunofluorescence analysis was done using the Micro Trak Chlamydia trachomatis Direct Specimen Kit procured from M/s Trinity Biotech, Ireland® as per manufacturer's instructions. In brief, all slides containing patient's specimens were fixed with absolute methanol, air dried, and stored at 0°C until they could be screened for Chlamydia trachomatis. A positive control and a negative control, as provided by the supplier, were processed along with each set of specimens to ensure reliability of the reagents. Morphology for positive specimens was confirmed at a magnification of 100x. All slides were screened for a minimum of 20 minutes. Specimens were considered positive only if a minimum of 10 smooth elementary bodies (indicative of Chlamydia trachomatis) were observed demonstrating fluorescence with a characteristic apple green refringence on the same plane as the conjunctival cell nucleus.

3.3.9 Information of Mass Drug Administration Coverage:

The information on Mass drug administration of the family members in the household was taken from the head of the household or any other responsible member in case the head of household was not available. The information was also collected for the members not available in the house at the time of survey. The records of Mass drug administration available with the sub-centres were also seen.

3.3.10 Data Analysis

The data was collected in semi-structured proformas and analyzed in STATA 12 software. The report and analysis was finalized by the Department of Community Ophthalmology, Dr. R.P. Centre for Ophthalmic Sciences, AIIMS, New Delhi.

3.3.11 Quality Assurance, Monitoring and Technical Advisory Group

An expert advisory group was formed before the start of the trachoma survey in Andaman & Nicobar Islands. This included key officials from the National Programme for Control of Blindness, Nirman Bhawan, Chief of R P Centre, AIIMS, epidemiological and ophthalmic experts from Department of Community Ophthalmology, RPC and State programme officer for Blindness Control. A central survey team constituting of the Chief Investigator, Epidemiologist & Senior Ophthalmologists supervised the field operations to ensure quality, analyze the data and prepared the survey report.

3.3.12 Ethical Approval

Ethical approval was taken from the Ethics Committee, AIIMS, New Delhi. Written informed consent was taken from head of the family or in case of his absence from any other adult member in the family. Each participant was informed about the study details using Participant Information Sheet.

3.4. Results

The island of Car Nicobar comprises of 15 villages with a total population of 20,294 (Census 2011). The prevalence survey was conducted in all the villages. In each village, one cluster was randomly identified with population ranging from 250 to 350 population. It was ensured that minimum 50 children in 1-9 years age group should be covered in each cluster for assessment of active infection.

The study population and clusters covered in the districts are shown in Table 1. A total of 4178 people were enumerated in all 15 clusters ranging from 182 in Arong to 382 in Maacca. The total number of people examined for trichiasis and corneal opacity due to trachoma was 2735 among the population 10 years and above. The coverage for ocular examination was 82.1% (ranging from 66.1% in Arong to 96.1% in Tapoiming). 809 children were examined for assessing signs of active

trachoma infection with coverage of 95.5% of the enumerated children (ranging from 90.9% to 100% in different clusters) The average number of children examined per village was 54 (Range = 50-66). More than half (57%) of the children examined were in the age group of 1-5 years.

The age and gender distribution of the study population in Table 2, showed that the proportion of females aged 10 years and above was higher (58.4%) but in contrast, the proportion of boys was more (52.4%) than girls amongst children aged 1-9 years.

Table 1: Study Population for Nicobar Trachoma Survey in Car Nicobar

Sl no.	Cluster	Population enumerated	> 10 years enumerated	> 10 years examined (96)	0-9 yrs enumerated	0-9 yrs examined {%)
1	KINYUKA	350	294	238 (81.0)	56	53 (94.6)
2	PERKA	221	165	132 (80.0)	56	54 (96.4)
3	TAMALU	273	213	168 (78.9)	60	58 (96.7)
4	MALACCA	382	324	268 (82.7)	58	54 (93.1)
5	SMALL LAPATHY	207	154	139 (90.3)	53	52 (98.1)
6	KINMAI	256	204	184 (90.2)	52	50 (96.2)
7	MUS	367	309	270 (87.4)	58	57 (98.3)
8	TEETOP	258	207	182 (87.9)	51	51 (100.0)
9	SAWAI	257	200	151 (75.5)	57	54 (94.7)
10	BIG LAPATHY	251	201	153 (76.1)	50	49 (98.0)
11	KAKANA	250	192	143 (74.5)	58	54 (93.1)
12	KIMIOUS	242	189	147 (77.8)	53	52 (98.1)
13	ARONG	182	123	82 (66.7)	59	54 (91.5)
14	TAPOIMING	317	257	247 (96.1)	60	57 (95.0)
15	CHUKCHUKA	365	299	231 (77.3)	66	60 (90.9)
	**Total**	**4178**	**3,331**	**2735 (82.1)**	**847**	**809 (95.5)**

Table 2: Age & Gender Distribution of Population Examined in Car Nicobar

S.No	Cluster	> 10 years			1-9 years		
		Male (%)	Female (%)	Total	Male (%)	Female (%)	Total
**1**	**KINYUKA**	**105 (44.1)**	**133 (55.9)**	**238**	**27 (50.9)**	**26 (49.1)**	**53**
**2**	**PERKA**	**50 (37.9)**	**82 (62.1)**	**132**	**24 (44.4)**	**30 (55.6)**	**54**
**3**	**TAMALU**	**51 (30.4)**	**117 (69.6)**	**168**	**31 (53.4)**	**27 (46.6)**	**58**
**4**	**MALACCA**	**122 (45.5)**	**146 (54.5)**	**268**	**27 (50)**	**27 (50)**	**54**
**5**	**SMALL LAPATHY**	**63 (45.3)**	**76 (54.7)**	**139**	**33 (63.5)**	**19 (36.5)**	**52**
**6**	**KINMAI**	**88 (47.8)**	**96 (52.2)**	**184**	**26 (52)**	**24 (48)**	**50**
**7**	**MUS**	**111 (41.1)**	**159 (58.9)**	**270**	**24 (42.1)**	**33 (57.9)**	**57**
**8**	**TEETOP**	**83 (45.6)**	**99 (54.4)**	**182**	**30 (58.8)**	**21 (41.2)**	**51**
**9**	**SAWAI**	**55 (36.4)**	**96 (63.6)**	**151**	**28 (51.9)**	**26 (48.1)**	**54**
**10**	**BIG LAPATHY**	**60 (39.2)**	**93 (60.8)**	**153**	**24 (49)**	**25 (51)**	**49**
**11**	**KAKANA**	**53 (37.1)**	**90 (62.9)**	**143**	**31 (57.4)**	**23 (42.6)**	**54**
**12**	**KIMIOUS**	**70 (47.6)**	**77 (52.4)**	**147**	**29 (55.8)**	**23 (44.2)**	**52**
**13**	**ARONG**	**25 (30.5)**	**57 (69.5)**	**82**	**24 (44.4)**	**30 (55.6)**	**54**
**14**	**TAPOIMING**	**113 (45.7)**	**134 (54.3)**	**247**	**32 (56.1)**	**25 (43.9)**	**57**
**15**	**CHUKCHUKA**	**89 (38.5)**	**142 (61.5)**	**231**	**34 (56.7)**	**26 (43.3)**	**60**
	**Total**	**1138 (41.6)**	**1597 (58.4)**	**2,735**	**424 (52.4)**	**385 (47.6)**	**809**

3.4.1 Prevalence of Active Infection in Car Nicobar

A total of 55 active trachoma cases were identified with a prevalence of 6.8% (95% CI 5.1- 8.5). The prevalence of active trachoma was more than 20% in two villages namely in Small Lapathy and Perka. Cases with active trachoma infection were seen in 12 villages except in Malacca, Mus and Big Lapathy. All the cases demonstrated follicular stage of trachoma, there was no case of trachomatous inflammation (TI) in the study population. A total of 42 children had unclean faces with a prevalence of 5.2% (95% CI 3.7- 6.7), the number ranging from 0-7 in the various villages. A significant association was observed between the active infection and unclean face among children. (P< 0.001)

Conjunctival swab could be collected from the eyes of 46 children with active trachoma infection. The microbiological investigations were processed at the Ocular Microbiology Department of Dr. R.P. Centre, AIIMS, NewDelhi. 39 samples were found positive (84.8%) by the Direct immunofluorescent assay for Chalamydia trachomatis.

All the children identified with active trachoma infection were given treatment with single dose of azithromycin.

Table 3: Distribution of Active Trachoma Infection in Car Nicobar Island

S.No	Cluster	0-9 year examined	0-9 year with TF	Prevalence of active Infection (%)	Number with Unclean Face	Prevalence of Unclean face (%)
1	KINYUKA	53	4	7.5	7	13.2
2	PERKA	54	12	22.2	5	9.3
3	TAMALU	58	3	5.2	2	3.4
4	MALACCA	54	0	0.0	1	1.9
5	SMALL LAPATHY	52	12	23.1	5	9.6
6	KINMAI	50	2	4.0	1	2
7	MUS	57	0	0.0	0	0
8	TEETOP	51	6	11.8	0	0
9	SAWAI	54	1	1.9	4	7.4
10	BIG LAPATHY	49	0	0.0	1	2
11	KAKANA	54	5	9.3	5	9.3
12	KIMIOUS	52	4	7.7	3	5.8
13	ARONG	54	3	5.6	2	3.7
14	TAPOIMING	57	1	1.8	1	1.8
15	CHUKCHUKA	60	2	3.3	5	8.3
	**Total**	**809**	**55**	**6.8**	**42**	**5.2**

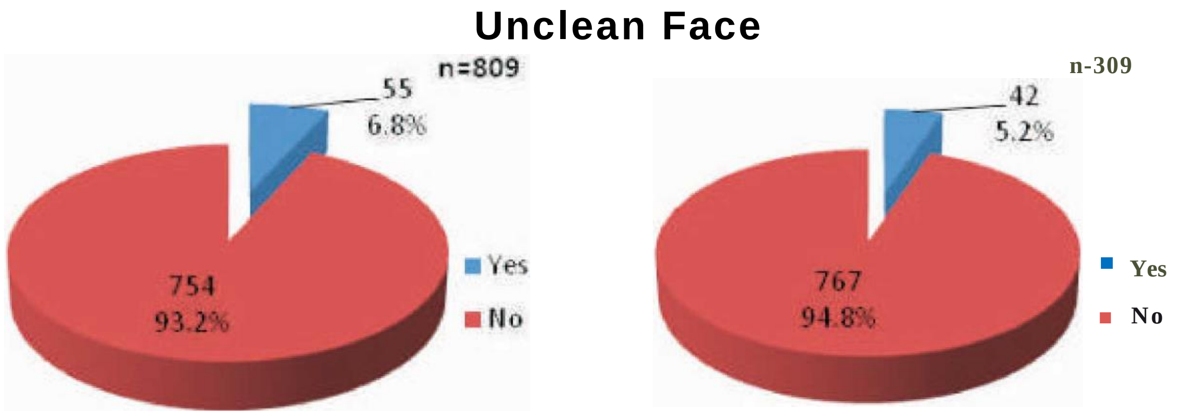

FiglA : Prevalence of active trachoma and B) unclean faces in children aged 1-9 years in Car Nicobar Islands

3.4.2 Prevalence of Trichiasis and Corneal Opacity in Car Nicobar Islands:

2735 people of the age 10 year and above were examined for trichiasis. Trichiasis cases were identified in all the 15 village clusters ranging from one case in Small Lapathy to 19 cases in Tapoiming. A total of 107 cases of trichiasis were found with a prevalence of 3.9%. Corneal opacity was found in 27% (29) cases with trichiasis. The prevalence of corneal opacity due to trachoma was 1.1% in the examined population. Nine patients had bilateral corneal opacity due to trichiasis. Three patients were blind (presenting visual acuity less than 6/60 in better eye) due to trachomatous corneal opacity. The list of all the trichiasis or corneal opacity cases was prepared and submitted to district administration so that surgical facilities may be arranged for these patients.

Table 4: Prevalence of Trichiasis in Car Nicobar islands

^S.No	Name of village	Households observed	Water Source distance more than half an hour walk (%)	Presence of solid waste or animal pens (%)	Absence of presence of^ functional latrine (%)
1	KINYUKA	56	1 (1.8)	49 (87.5)	0(0)
2	PERKA	35	0 (0.0)	31(88.6)	0(0)
3	TAMALU	37	0 (0.0)	35 (94.6)	0(0)
4	MALACCA	46	4 (8.7)	46 (100)	1 (2.2)
5	SMALL LAPATHY	21	0 (0.0)	15 (71.4)	0(0)
6	KINMAI	35	0 (0.0)	35 (100)	0(0)
7	MUS	43	0 (0.0)	42 (97.7)	1 (2.3)
8	TEETOP	31	1 (3.2)	22 (71)	0(0)
9	SAWAI	33	0 (0.0)	31 (93.9)	1(3)
10	BIG LAPATHY	38	0 (0.0)	38 (100)	0(0)
11	KAKANA	41	0 (0.0)	41 (100)	0(0)
12	KIMIOUS	39	0 (0.0)	24 (61.5)	0(0)
13	ARONG	26	0 (0.0)	26 (100)	0(0)
14	TAPOIMING	32	0 (0.0)	32 (100)	0(0)
15	CHUKCHUKA	39	1 (2.6)	28 (71.8)	1(2.6)
	**Total**	**552**	**7 (1.3)**	**495 (89.7)**	**4(0.7)**

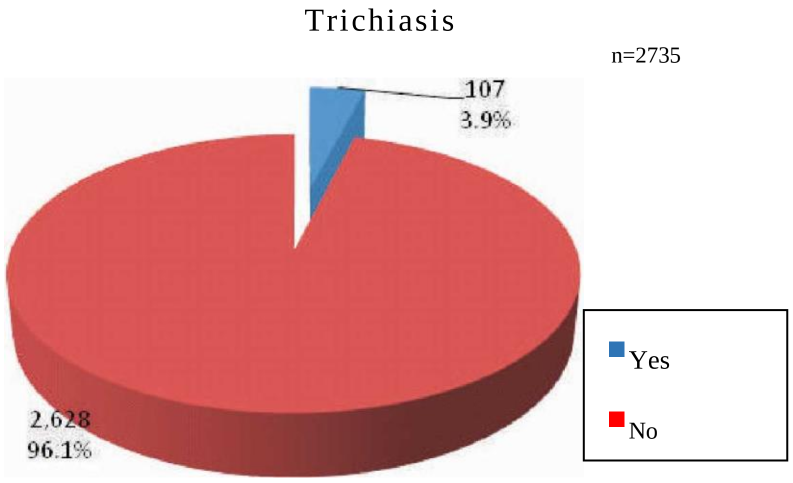

Fig 2: Prevalence of Trachomatous trichiasis in Car Nicobar Islands

3.4.3 Assessment of Environmental Risk factors in Car Nicobar Islands:

A total of 552 households in 15 clusters were observed for the environmental risk factors- the distance of source of water, presence of solid waste or animal pens and absence of functional latrines. Water source was not available within half an hour walking distance in only seven households (1.3%). Presence of solid waste or animals in & around the household was observed in majority of households (89.7%) Functional latrines were available in most of the households (99.3%). Environmental sanitation especially co-habitance of animals in and around the household is the major risk factor for trachoma in this island. There were all type of animals like pigs, hens, dogs, goats and cats. In many households more than 20 animals were also observed.

Table 5: Distribution of Households by Environmental Risk Factors in Car Nicobar

Sl.no	Cluster	10 years & & above examined	TT without without CO%	TT with CO%	Recurrent Trichiasis %	Total TT	Prevalence TT%
1	KINYUKA	238	5 (2.1)	4 (1.7)	0(0)	9	3.8
2	PERKA	132	2 (1.5)	1 (0.8)	0(0)	3	2.3
3	TAMALU	168	5 (3.0)	4 (2.4)	5 (3.0)	14	8.3
4	MALACCA	268	8 (3.0)	4 (1.5)	0(0)	12	4.5
5	SMALL LAPATHY	139	0 (0.0)	1 (0.7)	0(0)	1	0.7
6	KINMAI	184	6 (3.3)	1 (0.5)	0(0)	7	3.8
7	MUS	270	11 (4.1)	2 (0.7)	0(0)	13	4.8
8	TEETOP	182	4(2.2)	1 (0.5)	0(0)	5	2.7
9	SAWAI	151	2(1.3)	0 (0.0)	0(0)	2	1.3
10	BIG LAPATHY	153	1 (0.7)	4 (2.6)	0(0)	5	3.3
11	KAKANA	143	2 (1.4)	3 (2.1)	0(0)	5	3.5
12	KIMIOUS	147	1 (0.7)	0 (0.0)	0(0)	1	0.7
13	ARONG	82	2 (2.4)	0 (0.0)	0(0)	2	2.4
14	TAPOIMING	247	14 (5.7)	4 (1.6)	1 (0.4)	19	7.7
15	CHUKCHUKA	231	9 (3.9)	0 (0.0)	0(0)	9	3.9
	**Total**	**2,735**	**72 (2.6)**	**29 (1.1)**	**6(0.2)**	**107**	**3.9**

3.4.4 Access to Facilities in Car Nicobar

The facilities for trichiasis surgery were not available in Car Nicobar, as the state has ophthalmologists posted only at Port Blair. All the villages have access to primary eye care facility, village pharmacy (in the subcentre) and school within walking distance of less than 30 minutes. But in three villages namely Small Lapathy, Kakana and Arong, it was reported that the access to market place was more than 30 minutes of walk. Facilities for surgical management of trachoma sequalae & its complications were not available in the district. The surgical facilities for

Table 6: Access to facilities in Car Nicobar

fS.No	Cluster	Primary Health Care facility	Trichiasis surgery facility	Village Pharmacy (Drug Store)	Market	School
1	KINYUKA	1	3	1	1	1
2	PERKA	1	3	1	1	1
3	TAMALU	1	3	1	1	1
4	MALACCA	1	3	1	1	1
5	SMALL LAPATHY	1	3	1		1
6	KINMAI	1	3	1	1	1
7	MUS	1	3	1	1	1
8	TEETOP	1	3	1	1	1
9	SAWAI	1	3	1	1	1
10	BIG LAPATHY	1	3	1	1	1
11	KAKANA	1	3	1		1
12	KIMIOUS	1	3	1	1	1
13	ARONG	1	3	1		1
14	TAPOIMING	1	3	1	1	1
15	CHUKCHUKA	1	3	1	1	1

trachoma were arranged through camp approach by the ophthalmologist from Port Blair.

Distance to Facility: <30min=1; 30min-2hr=2; >2hr=3

3.4.5 Coverage for Mass Drug Administration in Car Nicobar

Mass drug administration was given in all the villages for three consecutive years in 2010, 2011 and 2012. ASHA and Anganwadi workers distributed the drug in their respective villages. Records for coverage of MDA were observed at the subcentres as well as at State NPCB office in Port Blair. The households covered with MDA were marked along with the year of MDA. Survey team collected information for MDA from the household respondents. The information may not be very reliable due to language barrier and low awareness among the population about

trachoma. Mass drug administration for other diseases like malaria and filariasis was also given during the recent period. People mentioned about the drug administration but they were not able to provide information specific to MDA for trachoma. The estimated coverage was more than 80% in all the three years.

Table 7: Coverage for Mass Drug Administration in Car Nicobar

S.No	Cluster	Eligible population for MDA 2012 (population >2 years) *	MDA in 2012(%)	Eligible population for MDA 2011 (population >3 years) **	MDA in 2011(%)	Eligible population for MDA 2010 (population >4 years) ***	MDA in 2010 (%)
1	KINYUKA	337	280 (83.1)	333	256 (76.9)	324	216 (66.7)
2	PERKA	212	174 (82.1)	205	156 (76.1)	197	150 (76.1)
3	TAMALU	261	246 (94.3)	258	245 (95.0)	255	242 (94.9)
4	MALACCA	367	352 (95.9)	360	340 (94.4)	351	325 (92.6)
5	SMALL LAPATHY	195	172 (88.2)	188	164 (87.2)	179	157 (87.7)
6	KINMAI	245	242 (98.8)	241	225 (93.4)	238	214 (89.9)
7	MUS	357	267 (74.8)	350	238 (68.0)	342	186 (54.4)
8	TEETOP	249	218 (87.6)	244	195 (79.9)	238	179 (75.2)
9	SAWAI	246	245 (99.6)	238	235 (98.7)	233	232 (99.6)
10	BIG LAPATHY	240	204 (85.0)	231	201 (87.0)	227	195 (85.9)
11	KAKANA	240	239 (99.6)	231	229 (99.1)	224	203 (90.6)
12	KIMIOUS	233	189 (81.1)	224	180 (80.4)	212	171 (80.7)
13	ARONG	174	169 (97.1)	164	158 (96.3)	155	118 (76.1)
14	TAPOIMING	307	300 (97.7)	298	283 (95.0)	290	268 (92.4)
15	CHUKCHUKA	347	319 (91.9)	336	311 (92.6)	329	301 (91.5)
	**Total**	**4010**	**3616 (90.2)**	**3901**	**3416 (87.6)**	**3794**	**3157 (83.2)**

3.4.6 Knoqledge about Trachoma in Car Nicobar:

One adult respondent in the family was asked questions related to knowledge & preventive measures related to trachoma. Trachoma is commonly known as ‘Chakoma’ in Car Nicobar. Only 307 (55.6%) respondents informed that they had ever heard about trachoma. Those who had heard of trachoma were asked about risk factors for spread of trachoma. Only 57 (10.7%) participants could answer at least one risk factor correctly that can lead to trachoma. The respondents were asked leading questions related to risk factors and preventive measures for trachoma. The knowledge among the participants was not satisfactory as shown in Table 8.

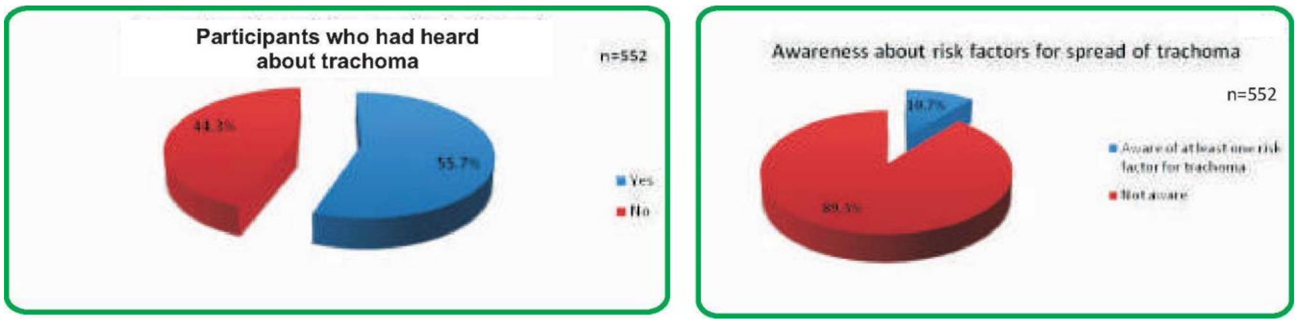

Fig 4A: Awareness about trachoma and B. risk factors responsible for spread of trachoma amongst the study population

Table 8: Knowledge about Trachoma in Car Nicobar

/- Question	Response ^ n=552 (%)
Have you heard about the disease named Trachoma (Chakoma)?	307 (55.6)
Can it spread by lack of hygienic measures?	267 (48.4)
Can it spread by contact with flies?	279 (50.5)
Can it spread by contact with animals?	287 (52.0)
Can it spread by overcrowding?	229 (41.5)
Can it spread by open sewer?	238 (43.1)
Can facewashing prevent trachoma?	266 (48.2)
Can handwashing prevent trachoma?	265 (48.0)
Can availability of functional latrine in the house prevent trachoma?	216 (39.1)
Can trachoma lead to blindness?	257 (46.6)
Can timely treatment of trachoma with antibiotics prevent its sequalae that leads to blindness?	221 (40.0)

3.4.7 Comparison of Trachoma status in 2010 and 2013 in Car Nicobar

In 2010, Rapid assessment for trachoma was conducted in 10 villages of Car Nicobar and active infection (TF/TI) was observed in 50.8% of the examined children ranging from 37.5% to 73.0% in different clusters. 251 children had evidence of follicular stage (TF) and another 11 had inflammatory stage (TI) of trachoma. The current survey showed significant reduction in the burden of trachoma infection in Car Nicobar and magnitude of active infection reduced to 6.8% among the examined children. There was no case of Trachoma inflammation intense (TI) in the examined population.

Table 9: Comparison of Active Infection in Car Nicobar in 2010 & 2013

	Rapid Assessment 2010			Prevalence Survey 2013		
**Clusters**	**No. Children Examined**	**No. With TF/TI**	**% Children With TF/TI**	**No. Children Examined**	**No. With TF/TI**	**% Children With TF/TI**
**KINYUKA**	**52**	**38**	**73.0**	**53**	**4**	**7.5**
**CHUKCHUCHA**	**54**	**24**	**44.4**	**60**	**2**	**3.3**
**ARONG**	**50**	**23**	**46.0**	**54**	**3**	**5.6**
**TAMALOO**	**52**	**23**	**44.2**	**58**	**3**	**5.2**
**KAKANA**	**53**	**24**	**45.3**	**54**	**5**	**9.3**
**BIG LAPATHY**	**50**	**29**	**58.0**	**49**	**0**	**0.0**
**TAPOIMING**	**52**	**27**	**51.9**	**57**	**1**	**1.8**
**SMALL LAPATHY**	**52**	**31**	**59.6**	**52**	**12**	**23.1**
**MUS**	**48**	**18**	**37.5**	**57**	**0**	**0.0**
**KINMAI**	**53**	**25**	**47.2**	**50**	**2**	**4.0**
**PERKA**				**54**	**12**	**22.2**
**MALACCA**				**54**	**0**	**0.0**
**TEETOP**				**51**	**6**	**11.8**
**SAWAI**				**54**	**1**	**1.9**
**KIMIOUS**				**52**	**4**	**7.7**
**TOTAL**	**516**	**262**	**50.8**	**809**	**55**	**6.8**

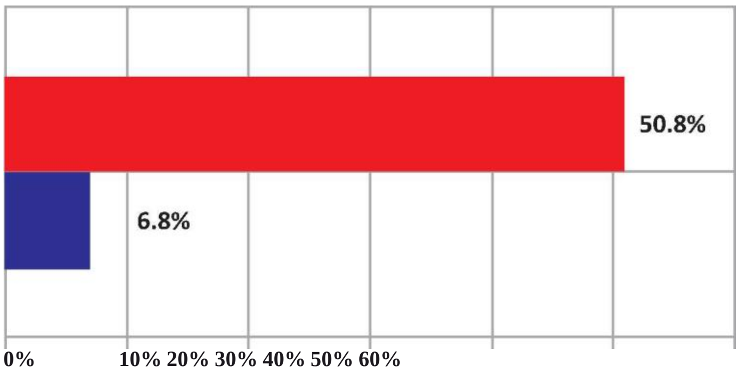

Fig 5. Comparison of burden of active trachoma infection in 2010 and 2013

3.5. Conclusion & Recommendations

The survey finding showed that the burden of active trachoma infection in Car Nicobar has reduced significantly from 50.8% in 2010 as assessed by the Trachoma Rapid Assessment (TRA) to 6.8% in 2013. This shows impressive effort by the State Blindness Control Programme in

applying measures for trachoma control in the island. The coverage for Mass Drug Administration with azithromycin was more than 80% in three consecutive years 2010, 2011& 2012. The reduction of proportion of children with unclean faces to 5.2% from 14.9% indicates increased awareness about "clean facial hygiene" for trachoma prevention. It is recommended that the district administration should continue with the efforts for trachoma control in order to achieve elimination of trachoma infection in next three years. Following measures are recommended:

3.5.1 Case finding & referral through training of local health workers

It was observed that the patients with trichiasis and other complications of trachoma are not availing the services due to unawareness or lack of services in the island. It is necessary the local health workers- ANMs and ASHA workers should be trained to identify TT patients and should ensure their treatment by liaising with the ophthalmologist in the hospital or camps.

3.5.2 Case specific treatment with azithromycin for active infection

Though the prevalence of active trachoma infection is reduced to 6.8%, it is not yet eliminated from the Island. At this stage, there is no need for the mass treatment with azithromycin, but it is extremely important that all the active cases should be identified and treated with azithromycin in order to prevent spread of infection. The village health workers including ANM and ASHA workers should be sensitized about the disease. There is need for routine ocular examination of children to identify active trachoma cases and their timely management. The Optometrists should be posted for examination of children at regular interval in the village & schools for this purpose. Medicine for treatment for active infection should be available in the subcentres and base hospital. Treatment of the family members of the children with active trachoma infection is also recommended.

3.5.3 Provision of surgical services for trichiasis

The results of the survey showed that there is a high burden of trichiasis cases in this region and there is need for surgical facilities for the trichiasis and its sequel cases to prevent development of corneal blindness. It is recommended that trachoma surgical camps may be organized in the Island with the help of experienced surgeons from other parts of the country. The experienced surgeons should also train the local ophthalmologists and paramedical staff for trachoma surgery to ensure sustainability of the surgical facilities and follow up services.

3.5.4 Improving environmental risk factors

It was found that there the environment risk factor related to presence of solid waste & animals around the households are still persisting in the region and in absence of adequate intervention the trachoma may spread further. In the absence of adequate drainage facilities, the unclean water collection around the household may lead to serious health hazards mainly during the rainy seasons. There is no proper garbage disposal facility available anywhere in the island, which is an important health concern. The co-habitance of people with animals like pigs, hens, goats, dogs, cats etc. is suspected to be the major risk factor of trachoma spread in this community. Pets were observed in close vicinity of most of the households. The district administration should use effective health promotion tools for educating people to keep pet animals away from the households.

3.5.5 Strengthening health promotion and preventive measures

Prevention of trachoma infection and promotion of health standards should be continued. It was identified that the knowledge about trachoma, its risk factors and preventive measures is still low in the population of Car Nicobar. The people should be educated about trachoma and how it is spread, encouraging acceptance for surgery and antibiotic treatment, encouraging facial cleanliness and promoting clean environment. The most appropriate channel in this region is the health promotion in community meetings under the leadership of village captains. There is still need for promotinginter- personal communication for discussing sensitive issues such as explaining need for behavioral changes, keeping pet animals away from the households and encouraging acceptance for surgery or treatment. The mass media like television and radio should be used for conveying discrete messages like dates of camp for trachoma surgery. It is suggested that a variety of IEC materials like posters or hoardings conveying simple messages for prevention of trachoma may be displayed in commonly visited areas like hospitals, sub- centres, schools, churches etc

Abbreviations

NPCB :	National Programme for Control of Blindness
ASHA :	Accredited Social Health Activist
AIIMS :	All India Institute of Medical Sciences
RPC :	Rajendra Prasad Centre for Ophthalmic Sciences
DGHS :	Directorate General of Health Services
IAS :	Indian Administrative Service
ANM :	Auxiliary Nurse Midwife
TF :	Trachoma Follicular
TI :	Trachoma Inflammation
TT :	TrachomatousTrichiasis
CO :	Corneal Opacity
SAFE :	Surgery, Antibiotics, Facial cleanliness, Environmental modification
WHO :	World Health Organization
TRA :	Trachoma Rapid Assessment
MDA :	Mass Drug Administration
PHC :	Primary Health Centre

Annexure - 1

TOUR DETAILS FOR TRACHOMA SURVEY IN A&N ISLANDS

1. Schedule for the RPC team

Date	Survey Programme	Team members from R.P.Centre
**March 28& 29, 2013**	Training of Ophthalmologists and other team members at Dr. R.P.Centre, AIIMS	Dr. Praveen Vashist, Dr. Sumit Malhotra, Dr. Noopur Gupta, Dr. Saurabh Agrawal, Dr. Babulal, Mr. Gopal Saha, Mr. Sandeep Kumar & Mr. Jaman Singh Rawat
**March 30, 2013**	Travel of RPC team to Port Blair, Andaman & Nicobar and training of GB Pant, Hospital team, A& N	
**March 31, 2013**	Combined survey in a village in Port Blair by both teams and then departure of team B to Mayabunder	Team A: Dr. Praveen Vashist, Dr. Noopur Gupta, Dr. Babulal, Mr. Sandeep<br>Team B: Dr. Sumit Malhotra, Dr. Saurabh Agrawal, Mr. Gopal Saha & Mr. Jaman Singh Rawat
**April 1, 2013 to April 4, 2013**	Tracoma Rapid Assessment in Port Blair by Team A (April 1-3, 2013) and Mayabunder (April 1-4, 2013) by team B	(details given in **table 1**)
**April 4, 2013**	Departure of Team A to Nicobar and training of local teams on Trachoma Prevalence Survey	Dr. Praveen Vashist, Dr. Noopur Gupta, Dr. Babulal, Mr. Sandeep
**April 5, 2013**	Departure of Prof. Azad &teamB to Nicobar Island and piloting	Dr. Sumit Malhotra, Dr. Saurabh Agrawal, Mr. Gopal Saha & Mr. Jaman Singh Rawat
**April 6, 2013 to April 10, 2013**	Survey in Nicobar Island	(details given in **table 2**)
**April 11, 2013**	Travel of R.P.C team from Nicobar to port Blair	Dr. Praveen Vashist, Dr. Sumit Malhotra, Dr. Noopur Gupta, Dr. Saurabh Agrawal, Dr. Babulal, , Mr. Gopal Saha, Mr. Sandeep Kumar & Mr. Jaman Singh Rawat
**April 12, 2013**	Return of members of R.P.C team back to Delhi	

2. Technical experts & monitoring team for the survey:

Ms. Sujaya Krishnan, JC, MOH&FW, Govt of India
Prof. Rajvardhan Azad- Chief, Dr. R.P.Centre for Ophthalmic Sciences, AIIMS
Dr. N.K.Agarwal, DDG, NPCB, Govt of India
Dr. Anita, SPO, Andman & Nicobar Island
Dr. Praveen Vashist, Additional Prof. & Head, Community Ophthalmology, Dr. R.P.Centre
Dr. Sumit Malhotra- Assistant Professor, Community Ophthalmology, Dr. R.P.Centre
Dr. Noopur Gupta -Ophthalmologist, Dr. R.P. Centre

3. Travel Plan of the R.P.Centre Team

1.All team members will leave on 30th March 2013 and will return on 12th April 2013.

Annexure - 1

Table 1: Trachoma Rapid Assessment in Port Blair and Mayabunder(5 Villages each)

Lead by:

Dr. Praveen Vashis t in Port Blair March 31, 2013 to April 3, 2013,

Dr. SumitMalhotra in Mayabunder district April 1, 2013 to April 4, 2013,

Designation	Team A- Portblair	Team B- Mayabunder
Ophthalmologist	Dr. Noopur Gupta, RPC& Dr. Babulal, RPC	Dr. Saurabh, RPC
Survey Supervisor	-	Mr.Jaman Singh Rawat, RPC
OptometristRPC	Mr. Sandeep, RPC	Mr. Gopal, RPC
Optometrist	Port Blair team	Mayabunder team
Field Attendant 1(6 days)	Port Blair team	Mayabunder team
Field Attendant 2 (6 days)	Port Blair team	Mayabunder team
Volunteer 1 (5 days)	Port Blair team	Mayabunder team
Volunteer 2 (5 days)	Port Blair team	Mayabunder team

Table 2: Trachoma prevalence study in Nicobar Island (15 villages)

April 5, 2013- April 10, 2013

Designation	Team A	Team B	Team C
Ophthalmologist	Dr. Noopur Gupta, RPC	Dr. Saurabh, RPC	Dr. Babulal, RPC
Survey Supervisor	From GB Pant Hospital	Mr. Jaman Singh Rawat, RPC	From GB Pant Hospital
Optometrist	Mr. Sandeep, RPC	From GB Pant Hospital	Mr. Gopal Saha, RPC
Field Investigator (7days)	From GB Pant Hospital	From Nicobar	From Nicobar
Field Attendant (7 days)	From Nicobar	From Nicobar	From Nicobar
Volunteer (7 days)	From Nicobar	From Nicobar	From Nicobar
Volunteer(7 days)	From Nicobar	From Nicobar	From Nicobar

Annexure - 2

Study Questionnaire

(D) Details of all adult members in the household (Start with oldest living member)

S. No	Relation with HOH	Age	Sex	Education	Occupation	Trichiasis: CO With	Trichiasis: CO Without	Trichiasis: Recurrent	Trichiasis: Suspect	Presenting V/A RE	Presenting V/A LE	Pinhole V/A RE	Pinhole V/A LE	Cause of V/A < 6/60 RE	Cause of V/A < 6/60 LE	Diagnosis if V/A <6/18 RE	Diagnosis if V/A <6/18 LE	History of MDA 2010 Y/N	History of MDA 2011 Y/N	History of MDA 2012 Y/N	CO due to other cause (Y/N)	if Yes then specify the cause
1																						
2																						
3																						
4																						
5																						
6																						
7																						
8																						
9																						
10																						

(E) Children <10 yrs

S. No	Relation with HOH	Age	Sex	Active Trachoma TF	Active Trachoma TI	Unclean Face Y/N	Stage if Vitamin A Deficiency
1							
2							
3							
4							
5							
6							
7							
8							

Xerophthalmia:
01. Night blindness(XN)
02. Conjuctival xerosis(X1A)
03. Bitot's Spot(X1B)
04. Corneal Xerosis(X2)
05. Corneal Ulceration <1/3 (X3A)
06. Corneal Ulceration >1/3 (X3B)
07. Corneal Scar(XS)
08. Xerophthalmic Fundus(XF)

CO due to other Causes:
01. Infectious
02. Chemical injury
03. Mechanical injury (Trauma)
04. Immunological
05. Idiopathic
06. Others (Specify):_______

Education:
00. Illiterate,
50. Can read & write
1-12. Years of schooling
13. Above 12th std.
66. Others (specify)

Relation with HOH
01. HOH
02. Father
03. Mother
04. Brother
05. Sister
06. Son
07. Daughter
08. Grand Son
09. Grand Daughter
10. Daughter in law
11. Others:________

Occupation:
01. House work
02. Coconut farming
03. Fish farming
04. Business (Fish net / Nuts)
05. Animal Husbandry
06. Govt. Job
07. Unemployed
08. Retired/ Not working because of old age
09. Student
66. Others (specify)___________

Diagnosis :
01. Refractive error
02. Cataract, untreated
03. Aphakia, uncorrected
04. Surgical complications
05. Trachoma
06. Phthisis
07. Other corneal scar
08. Globe abnormality
09. Glaucoma
10. Diabetic retinopathy
11. ARMD
12. Onchocerciasis
13. Pseudophakia/Aphakia corrected
15. Other post. segment / CNS
16. Not examined (can see 6/18)

Trichiasis:
Recurrent case: person already operated on for trichiasis; however, at least one eyelash rubs on the eyeball.
Suspected case: person not examined by the team, but reported by family / neighbours to have eyelid/ eyelash problem.

Annexure - 3

Training workshop for Trachoma Surveys in A&N Island 28-29th March 2013 Venue: Community Ophthalmology Department 7th floor, Dr. R.P.Centre, AIIMS, New Delhi

Date & Time	Session	Speaker/ Facilitator
**28th March 201 L3**		
9.30-9.45	Address by Prof. Rajvarshan Azad, Chief, R.P.Centre	
9.45- 10.00	Trachoma magnitude and objectives for conducting survey in A&N	Dr. Praveen Vashist
10.00-10.20	Management of Trachoma at community level — SAFE Strategy	Dr. Sumit Malhotra
10.20-11.00	Who grading scheme and Clinical features of different stages of trachoma: Including demonstration of cases from OPD, equipment for clinical examination	Dr. Noopur Gupta
	**Trachoma Prevalence Study**	
11.00-11.20	Evaluation of trachoma — Prevalence study	Dr. Praveen Vashist
11.20-12.00	Data collection forms- Prevalence study	Dr. Sumit Malhotra
12.00-1.00	Grading of trachoma slide sets and agreement analysis	Dr. Praveen Vashist Dr. Noopur Gupta
1.00-2.00	Lunch	
2.00-3.00	Enumeration procedures, mapping village and available facilities & resources for trachoma prevalence	Dr. Praveen Vashist Dr. Sumit Malhotra
3.00-3.30	Microbiological & biochemical tests	Prof.. Geeta Satpathy Dr. Jasbeer Kaur
3.30 -4.00	Role and responsibilities, Logistic arrangements- check list	Dr. Praveen Vashis t Dr. Saurabh
4.00-5.15	Practical training on forms and study procedures for prevalence study	Dr. Praveen Vashist Dr. Sumit Malhotra Dr. Noopur Gupta
**2 9th March 2013**		
9.30-10.00	Feed back on forms filled by the team members	Dr. Sumit Malhotra Dr. Noopur Gupta
10.00-11.00	Presentation of day I agreement analysis, further training on slides, taking photographs	Dr. Praveen Vashist Dr. Noopur Gupta
	**Trachoma Rapid Assessment**	
11.00-11.20	Evaluation of trachoma — Rapid Assessment method	Dr. Praveen Vashist
11.20-12.00	Data collection forms- for rapid Assessment	Dr. Sumit Malhotra
12.00-1.00	Rapid Assessment-Identifying village leaders and segmenting villages, enumeration method, Key informants Interview, mapping village facilities & resources, completion of environmental factors forms, completion of RA summary sheets,	Dr. Praveen Vashist Dr. Sumit Malhotra
1.00-2.00	Lunch Break	
2.00-4.00	Practical training- on forms, survey procedures- RA and also on Prevalence study	D r. Praveen Vashis t Dr. Sumit malhotra Dr. Noopur Gupta
4.15-5.15	Presentation on practical training and discussions	Dr. Praveen Vashist Dr. Sumit Malhotra Dr. Noopur Gupta
